# Evaluation of Microincision Vitrectomy Surgery Using Wide-Viewing System for Complications With Ocular Sarcoidosis

**DOI:** 10.1097/MD.0000000000000559

**Published:** 2015-02-20

**Authors:** Kei Takayama, Atsushi Tanaka, Masanori Shibata, Tadashi Muraoka, Sho Ishikawa, Kouzo Harimoto, Masaru Takeuchi

**Affiliations:** From the Department of Ophthalmology, National Defense Medical College.

## Abstract

We evaluate the outcomes of microincision vitrectomy surgery (MIVS) using wide-viewing system for complications with ocular sarcoidosis resistance to medical treatment.

Consecutive clinical records of 24 eyes (19 patients) with complications of ocular sarcoidosis underwent MIVS between April 2010 and December 2013 were retrospectively reviewed. MIVS and phacoemulsification were performed in 18 eyes and MIVS only in 6 eyes. Best-corrected visual acuity (BCVA), inflammation scores in the anterior segment and in the posterior segment, and central retinal thickness (CRT) of eyes with cystoid macular edema (CME) before surgery and after 1 week, 1, 3, 6, and 12 months were evaluated.

LogMAR (log of the minimum angle of resolution) converted from BCVA was improved in 83.3% after 12 months and 66.7% showed improvement of more than 2 lines. The mean LogMAR was significantly improved from 1.14 ± 1.18 to 0.36 ± 0.79 in all eyes and 0.83 ± 0.86 to 0.23 ± 0.41 in eyes with MIVS and phacoemulsification, although no improvement was observed in eyes with MIVS only. Significant decrease of the mean anterior inflammation score was observed after 1 month in eyes with MIVS only and after 12 months in eyes with MIVS and phacoemulsification, and the mean posterior inflammation scores decreased after 1 week in all eyes. In eyes with preoperative CME, mean CRT was significantly decreased from 1 week after surgery. There was no case in which ocular inflammation was exacerbated by surgical stress.

Improvement of visual acuity and resolution of ocular inflammation could be achieved by MIVS using wide-viewing system for complications of ocular sarcoidosis.

## INTRODUCTION

Sarcoidosis is a chronic inflammatory disease characterized by forming noncaseating granulomas in multiple tissues and organs including the eye.^1,2^ The treatment of ocular sarcoidosis is primarily to reduce ocular inflammation by medications.^3,4^ Although chronic and recurrent inflammations give rise to irreversible complications refractory to medical treatment, surgical treatment is used to maintain or improve the patient's visual function.^5,6^

For complications in the posterior segment of the eye, pars plana vitrectomy was performed. Benefits of vitrectomy for eyes with uveitis are clearance of inflammatory factors accumulated in the vitreous, removal of fibrotic proliferation affecting visual functions, improvement of intraocular metabolism, and increase of retinal oxygen partial pressure.^6–8^ Conventional vitrectomy performed with 20-gauge instrumentation had been a valuable modality, promoting clearing of vitreous opacities and repair of structural complications.^9–12^ Despite these advantages, intraocular surgery could also exacerbate inflammation in eyes with uveitis by activating the underlying inflammatory process, and the procedure per se may evoke an unusually severe inflammatory response, abnormal bleeding, or unexpected postoperative intraocular pressure response.^11^ Microincision vitrectomy surgery (MIVS), a transconjunctival sutureless surgery, was developed in 2002.^13–15^ MIVS is less invasive than the conventional 20-gauge vitrectomy, because conjunctival and scleral incisions are not required and the 1-step trocar/cannula system reduces stress on the ciliary body caused by repeated insertion and extraction of surgical instruments.^16,17^ In addition, by combined use with a wide-viewing system, suture fixation of the contact lens ring and invasive scleral depression for shaving the vitreous base are no longer necessary.^18,19^ These advantages shorten surgical time and reduce tissue injury and infection risk, subsequently reducing postoperative intraocular inflammatory response and hastening postoperative recovery.^20^

The use of MIVS for treating complications associated with endogenous uveitis has been indicated^20–22^; however, there is no report limited to only those of ocular sarcoidosis. The purpose of the present study was to assess surgical outcome of MIVS using a wide-viewing system for the treatment of ocular sarcoidosis-associated complications refractory to medications.

## MATERIALS AND METHODS

### Patients

We reviewed the clinical records of consecutive cases in which MIVS with wide-viewing system was performed for complications of ocular sarcoidosis from July 2006 to October 2013. Twenty-four eyes of 19 patients were enrolled and the average age was 66.1 ± 9.9 years (ranging from 50 to 79 years). Male to female ratio was 9 to 15. MIVS combined with phacoemulsification (MIVS with phacoemulsification) was performed in 18 eyes and MIVS only in 6 eyes. The complications are summarized in Table 1. Including overlaps, the most frequent complications were cystoid macular edema and epiretinal membrane (ERM) in 6 cases, followed by vitreous hemorrhage in 4 cases. All patients enrolled in this study were followed for more than 12 months after MIVS. The study was approved by the institutional review board of National Defense Medical College.

**TABLE 1:**
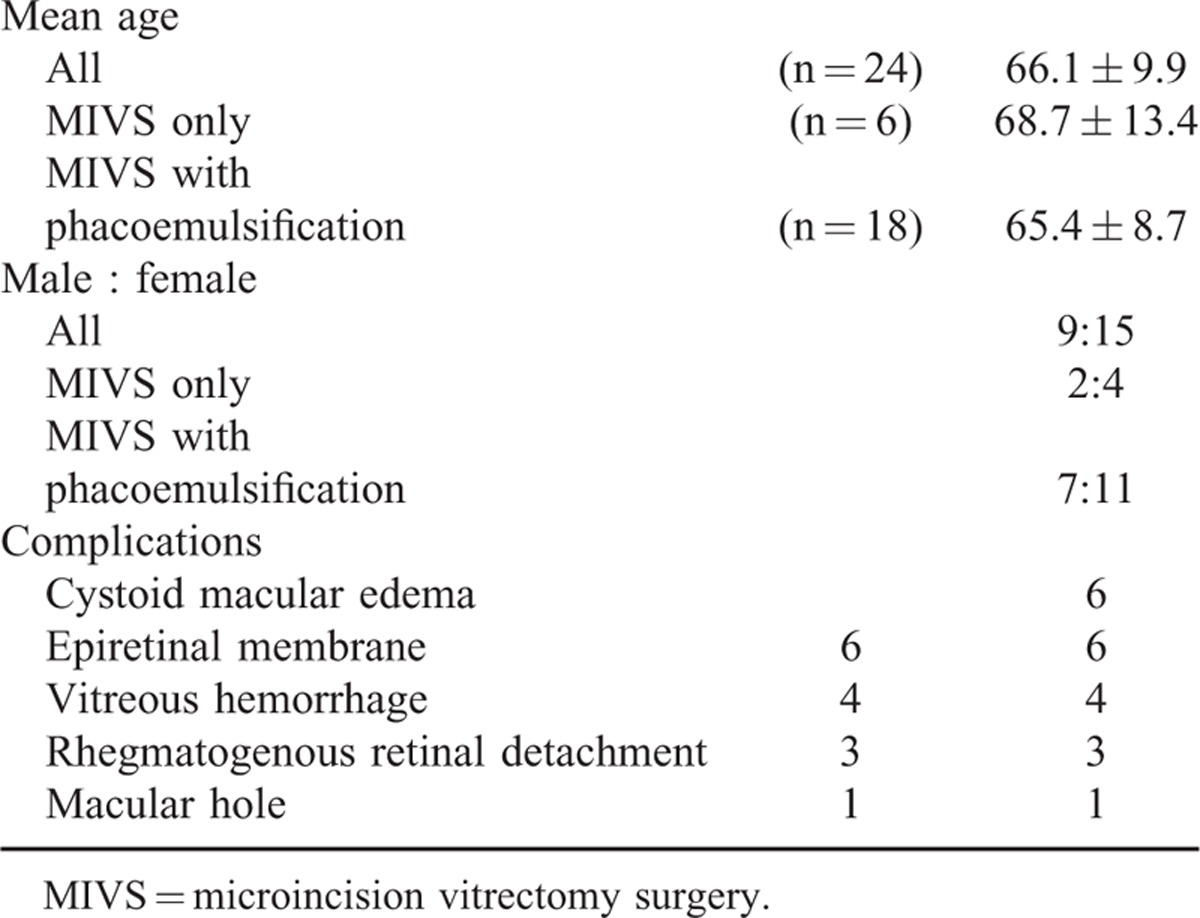
Patient Characteristics and Cause of Complications Treated for MIVS

### Surgical Techniques

After informed consent was obtained, the patients underwent surgery under local anesthesia. For patients with cataract, phacoemulsification was first performed with a 2.2-mm sutureless transconjunctival single-plane sclerocorneal incision at the 12-o’clock position, using an Infiniti Vision Systems phaco unit (Alcon Japan Co., Ltd., Tokyo, Japan). Foldable acrylic lens, SN60WF (Alcon, Inc) or NY-60 (HOYA Inc., Tokyo, Japan), were implanted in the bag with injectors after subsequent vitrectomy.

A 25-gauge microcannular system was used for vitrectomy. The microcannulae were inserted through the conjunctiva into the eye (3.5 mm posterior to the limbus). Vitrectomy using a wide viewing system (BIOM, Oculus, Wetzlar, Germany) was performed, and a disposable high reflective index vitrectomy meniscus contact lens (Hoya, Tokyo, Japan) was used only during macular surgery. Brilliant blue G-assisted inner limiting membrane peeling was performed in all eyes with ERM, cystoid macular edema, or macular hole. Scleral depression for shaving the vitreous base, endolaser photocoagulation, air exchange, and scleral buckling were performed as necessary. At the end of the vitrectomy, 4.0 mg of triamcinolone acetonide was injected intraocularly for eyes without steroid-induced glaucoma. After vitrectomy, systemic medication was continued, and eye drops of 0.1% betamethasone sodium phosphate and 0.5% gatifloxacin hydrate were provided 4 times per day and bromfenac sodium hydrate was 2 times per day for 2 months. Thereafter, eye drops used before surgery were restarted. Medications administrated before vitrectomy were continued during the surgical period. Systemic medications were initiated or increased according to the grade of ocular inflammation after surgery, and was discontinued when postoperative inflammation was resolved. Patients usually had follow-up evaluations at day 7 and every month thereafter. At each follow-up, complete ophthalmic examination was performed, with additional fluorescein angiography and optical coherence tomography when required.

### Outcome and Data Analysis

The best-corrected visual acuity (BCVA) and ocular inflammation scores in the anterior segment and posterior segment in the remission phase before surgery and at the 1 week, 1, 3, 6, and 12 months after MIVS were used for analysis. Ocular inflammation based on the degrees of cells and flare in the anterior segment and posterior segment were scored on a scale of 0 to 4 as reported by Nussenblatt et al ^23^ In eyes with preoperative macular edema, central retinal thickness before surgery and at 1 week, 1, 3, 6, and 12 months were used for analysis. The outcomes were analyzed statistically by non-repeated measure analysis of variance (ANOVA). *P* < 0.05 was considered to be significant.

## RESULTS

### Outcomes of Visual Acuity

**FIGURE 1:**
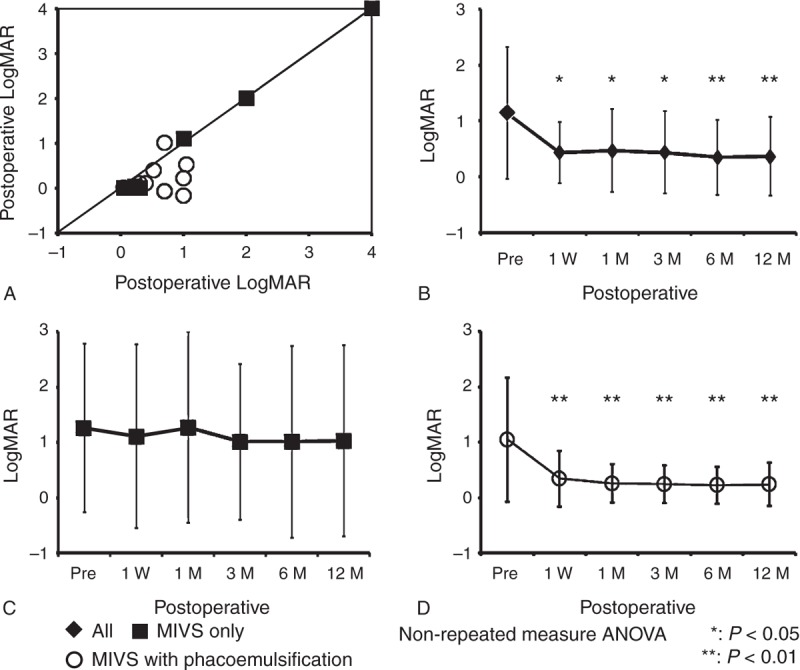
Outcomes of visual acuity before and after MIVS. A: LogMAR converted from BCVA in individual eyes of sarcoidosis patients before surgery and at 12 months after surgery. B, C, and D: The mean LogMAR before surgery and at 1 week, 1, 3, 6, and 12 months after surgery in all eyes (B), eyes with MIVS only (C), and eyes with MIVS and phacoemulsification (D). ANOVA = analysis of variance, LogMAR = log of the minimum angle of resolution, MIVS = microincision vitrectomy surgery.

The BCVA converted to LogMAR (log of the minimum angle of resolution) before surgery and after 1 week, 1, 3, 6, and 12 months is shown in Figure 1. Visual acuity was improved in 20 (83.3%) of 24 eyes after 12 months, 16 eyes (66.7%) showed improvement of more than 2 lines, whereas 4 eyes (16.7%) showed worsened or unchanged visual acuity (Figure 1A). The mean LogMAR in all eyes decreased from 1.14 ± 1.18 before surgery to 0.43 ± 0.55 at 1 week, 0.47 ± 0.74 at 1 month, 0.43 ± 0.73 at 3 months, 0.35 ± 0.67 at 6 months, and 0.36 ± 0.79 at 12 months after surgery, and significant improvements (*P* = .0028) were observed from 1 week (Figure 1B). When the cases were classified into MIVS only group and MIVS with phacoemulsification group, the mean LogMAR in MIVS only group was 1.26 ± 1.52 before surgery, and changed to 0.86 ± 0.92 at 1 week, 1.07 ± 1.35 after 1 month, 1.24 ± 1.51 at 3 months, 1.02 ± 1.72 at 6 months, and 1.03 ± 1.40 at 12 months (Table 2). There were no significant differences (*P* = .47) (Figure 1C). In MIVS with phacoemulsification group, the mean LogMAR was 1.05 ± 1.12 before surgery, changed to 0.34 ± 0.51 at 1 week, 0.26 ± 0.35 at 1 month, 0.23 ± 0.36 at 3 months, 0.21 ± 0.35 at 6 months, and 0.23 ± 0.41 at 12 months, and significantly decreased (*P* = .0027) after 1 week (Figure 1D).

**TABLE 2:**
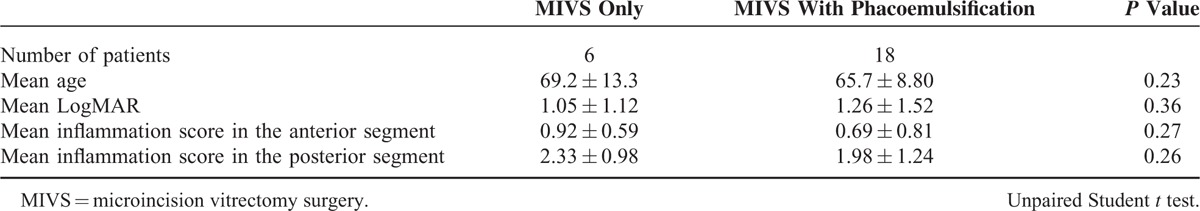
Patient Characteristics of MIVS Only and MIVS With Phacoemulsification

### Outcomes of Inflammatory Scores

**FIGURE 2:**
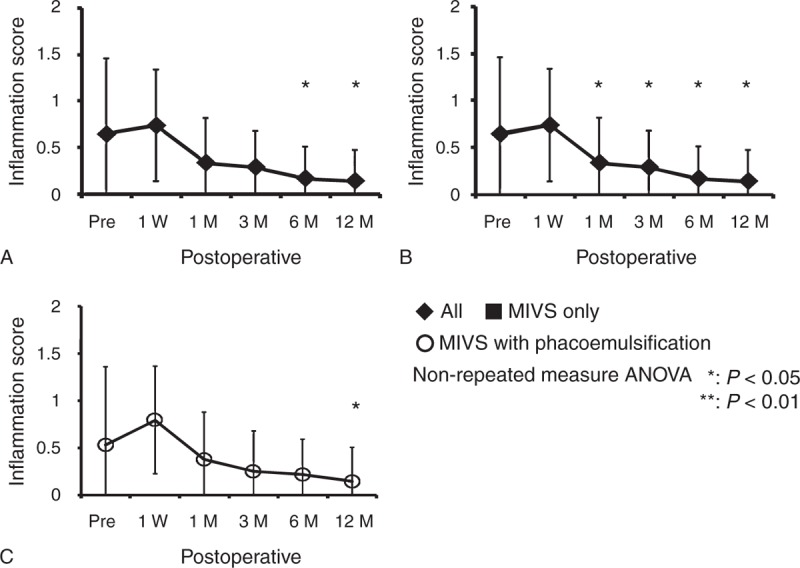
Outcomes of inflammatory scores in the anterior chamber. The inflammatory scores in the anterior chamber before surgery and at 1 week, 1, 3, 6, and 12 months after surgery in all eyes (A), eyes with MIVS only (B), and eyes with MIVS and phacoemulsification (C). ANOVA = analysis of variance, MIVS = microincision vitrectomy surgery.

Mean inflammation scores before surgery and at 1 week, 1, 3, 6, and 12 months after surgery were shown in Figures 2 (anterior segment) and 3 (posterior segment). In the anterior segment, mean inflammation score in all eyes before surgery was 0.65 ± 0.81, changed to 0.74 ± 0.60 at 1 week, 0.33 ± 0.48 at 1 month, 0.29 ± 0.38 at 3 months, 0.17 ± 0.34 at 6 months, and 0.14 ± 0.33 at 12 months, and significantly decreased (*P* *<* .001) at 12 months after surgery (Figure 2A). In MIVS only group, mean inflammation score before surgery was 0.91 ± 0.71, changed to 0.75 ± 0.61 at 1 week, 0.17 ± 0.45 at 1 month, 0.16 ± 0.22 at 3 months, 0.08 ± 0.22 at 6 months, and 0.17 ± 0.27 at 12 months, significantly decreased (*P* = .0049) after 1 month (Figure 2B). In MIVS with phacoemulsification group, mean inflammation score before surgery was 0.53 ± 0.83, changed to 0.79 ± 0.57 at 1 week, 0.38 ± 0.50 at 1 month, 0.25 ± 0.43 at 3 months, 0.21 ± 0.38 at 6 months, and 0.14 ± 0.36 at 12 months, and significantly decreased (*P* = .0037) at 12 months (Figure 2C).

**FIGURE 3:**
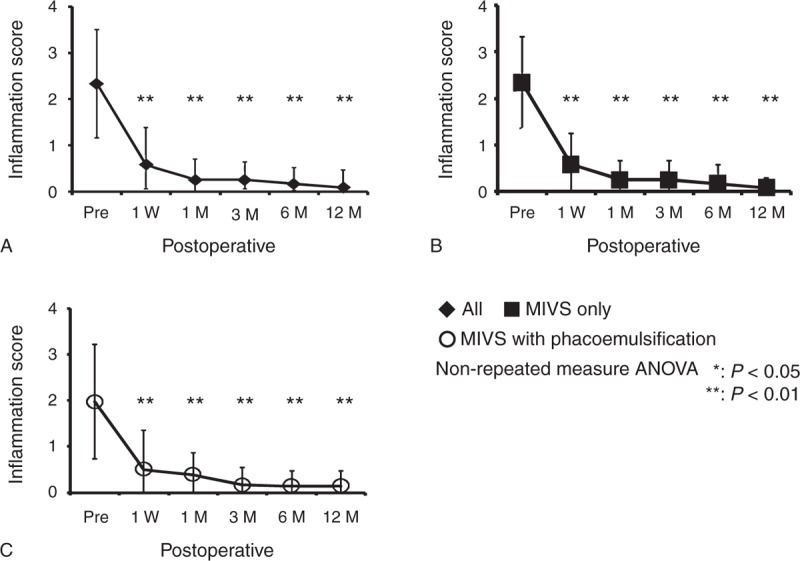
Outcomes of inflammation scores in the posterior chamber. The inflammatory scores in the posterior chamber before surgery and at 1 week, 1, 3, 6, and 12 months after surgery in all eyes (A), eyes with MIVS only (B), and eyes with MIVS and phacoemulsification (C). ANOVA = analysis of variance, MIVS = microincision vitrectomy surgery.

In the posterior segment, mean inflammatory score in all eyes before surgery was 2.06 ± 1.18, changed to 0.52 ± 0.80 at 1 week, 0.35 ± 0.45 at 1 month, 0.19 ± 0.38 at 3 months, 0.15 ± 0.35 at 6 months, and 0.19 ± 0.38 at 12 months, and significantly decreased (*P* *<* .001) from 1 week after surgery (Figure 3A). In MIVS only group, mean inflammation score before surgery was 2.33 ± 0.98, and decreased to 0.58 ± 0.66 at 1 week, 0.25 ± 0.42 at 1 month, 0.25 ± 0.42 at 3 months, 0.17 ± 0.41 at 6 months, and 0.08 ± 0.20 at 12 months (*P* *<* .001) (Figure 3B). In MIVS with phacoemulsification group, mean inflammation score before surgery was 1.97 ± 1.24, and decreased to 0.50 ± 0.86 at 1 week, 0.39 ± 0.47 at 1 month, 0.17 ± 0.38 at 3 months, 0.14 ± 0.33 at 6 months, and 0.14 ± 0.34 at 12 months (*P* *<* .001) (Figure 3C). Significant decrease of posterior inflammation was observed from 1 week after surgery in both MIVS only and MIVS with phacoemulsification.

### Outcome of Eyes With Cystoid Macular Edema

**FIGURE 4:**
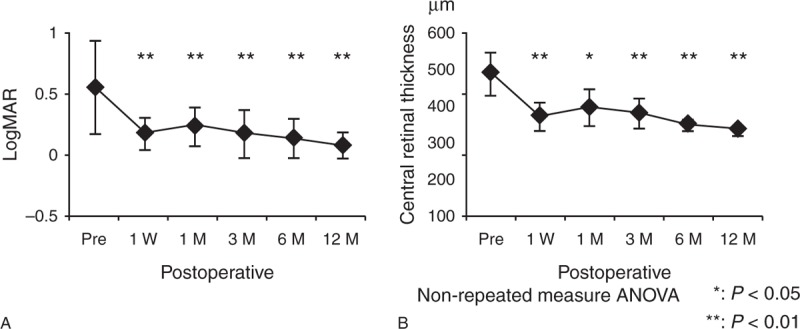
Outcome of eyes with cystoid macular edema before and after MIVS. The mean LogMAR in eyes with preoperative cystoid macular edema before surgery and at 1 week, 1, 3, 6, and 12 months after surgery (A), the mean central macular thickness in eyes with preoperative cystoid macular edema before surgery and at 1 week, 1, 3, 6, and 12 months after surgery (B). ANOVA = analysis of variance, LogMAR = log of the minimum angle of resolution.

The mean LogMAR in eyes with preoperative cystoid macular edema decreased from 0.56 ± 0.38 before surgery to 0.21 ± 0.12 at 1 week, 0.22 ± 0.16 at 1 month, 0.17 ± 0.19 at 3 months, 0.13 ± 0.16 at 6 months, and 0.07 ± 0.11 at 12 months after surgery (Figure 4A). Significant improvements of LogMAR were observed in these eyes from 1 week after surgery (*P* *<* .001). As corresponding to these results, the mean central macular thickness was decreased from 461 ± 71 μm before surgery to 321 ± 46 μm at 1 week, 351 ± 60 μm at 1 month, 333 ± 49 μm at 3 months, 294 ± 23 μm at 6 months, and 277 ± 21 μm at 12 months. Significant decrease of macular thickness compared with preoperation was observed from 1 week after surgery (*P* = .0071) (Figure 4B).

### Complications After Surgery

As postoperative complications, proliferative vitreoretinopathy in 1 eye, recurrence of vitreous hemorrhage in 1 eye, and rubeotic glaucoma in 1 eye were observed. Revitrectomy was applied for proliferative vitreoretinopathy and vitreous hemorrhage, and trabeculectomy combined with endolaser for pars plana was performed for rubeotic glaucoma.

## DISCUSSION

The present study indicates MIVS resolves ocular inflammation in sarcoidosis in addition to the improvement of visual acuity by removing complications resistant for medications. Accumulation of T cells with various cytokine profiles in the eye is thought to play a critical role in the pathogenesis of different types of chronic uveitis,^24,25^ and CD4/CD8 ratio of T cells infiltrating into the vitreous is elevated in ocular sarcoidosis patients compared with other uveitis patients as well as that in BAL. In addition, it has been reported that T-cell clones established from the vitreous T cells of ocular sarcoidosis patients produce a large amount of inflammatory cytokines including interleukin-1a, -6, -8, and that the production of these cytokines is not suppressed by corticosteroids.^26^ Since vitreous gel acts as a storage of these inflammatory cells and cytokines, removal of these factors and replacement with aqueous humor might lead to suppression of the ocular inflammation and to recovery of responsiveness for medications.

Conventional 20-gauge pars plana vitrectomy for ocular sarcoidosis were reported,^10,11^ in which visual acuity was improved in 63.6% of eyes with vitreous opacities, in 71.4% of eyes with CME, and in 55.6% of eyes with ERM. In the present study, 83.3% of all eyes improved visual acuity, 10 out of 12 (83.3%) eyes with CME and 8 out of 9 (88.9%) eyes showed improvement from 1 week after surgery. Although it is not proper to compare these results, it is likely MIVS using a wide-viewing system would be more suitable for the complications of ocular sarcoidosis than conventional 20-gauge pars plana vitrectomy. In idiopathic ERMs and rhegmatogenous retinal detachments, comparative outcomes of MIVS with 20-gauge pars plana vitrectomy were already reported. ^16,27–31^ MIVS improved visual acuity, anatomical reconstruction of the retina, and operative stress more than 20-gauge vitrectomy. They indicate MIVS is less invasive and may minimize postoperative inflammation.

However, visual acuity was improved in all eyes (18 eyes) when performed MIVS with phacoemulsification, while improvement of visual acuity was only 50% in eyes with MIVS only in which 33% showed no change, and 16.7% showed worsening. Anterior inflammation was higher in eyes performed MIVS with phacoemulsification than those of eyes with MIVS only during 6 months after surgery, but those were compatible after 12 months. Cataract progresses after vitreous surgery, which is accelerated by ocular inflammation, suggesting that combination with phacoemulsification is beneficial for improving visual acuity of eyes in which MIVS is performed for complications of ocular sarcoidosis.

Regarding 4 eyes (16.7%) which showed worsened or unchanged visual acuity after MIVS, unimproved macular edema was 2 eyes, proliferative vitreoretinopathy was 1 eye, and extensive retinal atrophy with rubeotic glaucoma was 1 eye. Since these complications had existed before surgery, it is considered that those were derived from the individual pathmechanisms of ocular sarcoidosis. There was no direct adverse effect such as exacerbated ocular inflammation by surgical stress or by removal of the vitreous.

In conclusion, the present study indicated that MIVS using wide-viewing system is effective for complications of ocular sarcoidosis resistant for medical treatment, by which remission of ocular inflammation and improvement of visual acuity are achieved.
